# Successful pregnancy after presurgical uterine artery embolization in the management of a very large cervical myoma: A case report

**DOI:** 10.1016/j.crwh.2022.e00450

**Published:** 2022-09-14

**Authors:** Suleyman Engin Akhan, Cenk Yasa, Ozlem Dural, Funda Gungor Ugurlucan, Izzet Rozanes

**Affiliations:** a Istanbul University School of Medicine, Department of Obstetrics and Gynecology, Istanbul, Turkey; b Koç University School of Medicine, Departments of Radiology, Istanbul, Turkey

**Keywords:** Cervical myoma, Uterine artery embolization, Pregnancy

## Abstract

Although fibroids are the most common benign tumors of the uterus in women of reproductive age, cervical fibroids are rarely seen. Since cervical fibroids are located deep in the pelvis, the incidence of complications in surgery is high. Among these complications bleeding is the most common, due to poor access to myoma, difficulty in suturing and repair, and distortion of vital neighboring structures. Each case should be managed individually to minimize bleeding. To decrease bleeding in patients who wish to retain their fertility, intraoperative interventions include vasoconstrictors such as vasopressin and adrenaline, uterotonics such as oxytocin, misoprostol or ergometrines, uterine artery clamping, internal iliac artery balloon occlusion catheters, and tourniquets; preoperative interventions include gonadotropin releasing-hormone analogues and uterine artery embolization. We present a case of a 40-year-old woman who had a large cervical myoma and a desire for future fertility. To overcome technical difficulties and reduce intraoperative bleeding during myomectomy, presurgical uterine artery embolization was performed. The patient conceived spontaneously after the operation and a healthy baby was delivered by cesarean section.

## Highlights

• Cervical myoma is rare.
• Preoperative uterine artery embolization decreases blood loss and helps preserve the uterus during myomectomy.
• In pregnant patients with a history of uterine artery embolization the risk of miscarriage, placental insertion anomalies, postpartum hemorrhage and intrauterine growth restriction is increased.

## 1 Introduction

Uterine myomas are the most common benign tumors in women. Myomas arising from the cervix are called cervical myomas; they are reported to account for 5% of all myomas [1]. They may cause menorrhagia, intermenstrual bleeding, pelvic pain, pressure effects and decreased fertility. Myomectomy is preferred to hysterectomy for the surgical treatment of myomas in women who wish to retain their fertility. Enlargement of the cervix due to myoma causes displacement of nearby structures such as the bladder, ureter and uterine blood vessels which makes them vulnerable during the surgery. Compared with myomas in the uterine corpus, cervical myomas present surgical difficulties such as poor access to the operative field, difficulty in suturing, distortion of anatomy and increased blood loss [2].

Controlling blood loss during myomectomy is challenging surgeons. Where there is the risk of heavy bleeding, the uterus may not be repaired and preserved. Preoperative measures to reduce blood loss include gonadotropin-releasing hormone analogues, aromatase inhibitors and selective progesterone receptor modulators. Presurgical uterine artery embolization (PUAE) has been offered to patients with large myomas to improve the surgical outcome and lower the risk of blood loss [3]. The most important concern related with PUAE is the risk of fertility loss and complications of pregnancy [4].

Here we present the case of a woman with a large cervical myoma managed by myomectomy after PUAE who went on to achieve a spontaneous pregnancy that resulted in the deliver of a healthy child.

## 2 Case presentation

A 40-year-old woman presented with complaints of pain and pressure sensation in the pelvic region. Her medical and surgical history was unremarkable except for two cesarean deliveries. During pelvic examination the external os of the cervix could not be clearly inspected by speculum due to posterior deviation of the cervix. On bimanual examination an immobile solid mass originating from the posterior wall of cervix and filling of the pouch of Douglas was palpated.

On magnetic resonance imaging, at the greatest dimension of the protrusion, an 11.5 cm fibroid nucleus was detected out of the left lateral posterior of the uterus originating from the cervical posterior stroma. In addition, while fibroids entered the bladder on the left and were pushing the ureter to the left, it partially contained the pseudocapsule near the bladder. The surgical team considered surgery without prior embolization to be too risky. The patient had a consultation with the radiology team. It was decided to perform PUAE and then myomectomy via laparotomy due to the diameter of the myoma. Possible risks and complications were explained to the patient and written informed consent was obtained.

**Fig. 1:**
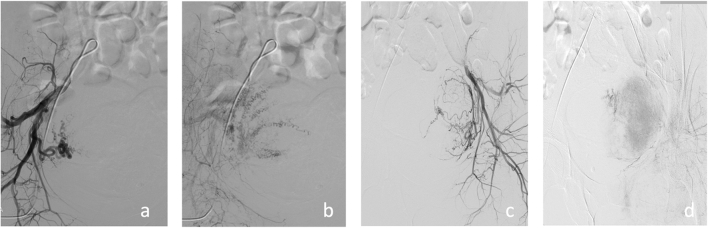
a) Left uterine artery arteriogram; b) after embolization of the left uterine artery with microspheres; c) right uterine artery arteriogram; d) embolization of the right uterine artery.

The patient was admitted and initially received bilateral uterine artery embolization after probing micro-catheter. Bilateral (20/21) femoral access was chosen, with the patient under sedation. To achieve near stasis, the type of particle used was microspheres (Embosphere, Meritmedical/Biosphere, Roissy, France), 500–700 μm, followed by 700–900 μm (Fig. 1 a-b-c-d). The procedure was technically successful; it took 15 min and there were no peri-procedural complications.

Twenty-four hours of after embolization, the patient had a longitudinal incision to permit the myomectomy. She did not require any analgesia during this period. Myomectomy was performed without damaging cervical integrity. There was no serious bleeding and no organ injuries. With total blood loss of 520 ml (aspirator content and estimate based on used sponges), there was a drop in serum hemoglobin from 13,4 g/dl preoperatively to 10,8 g/dl postoperatively. She did not need any blood transfusion. Total hospitalization time was 4 days and the postoperative period was uneventful. A benign leiomyoma was histologically confirmed. The patient resumed menstruation 40 days after surgery.

The patient conceived spontaneously at the 11th month after the operation. Screening exams and prenatal tests were negative. There were no complications related to the pregnancy in the antenatal period. At the 39th week, a healthy baby weighing 3240 g with a 9/9 Apgar score was delivered by cesarean delivery. The postoperative course was unremarkable.

## 3 Discussion

Uterine myoma can significantly impact a woman's health, fertility, and quality of life. Myomectomy is the surgical procedure that aims to preserve fertility while treating patients with symptomatic myoma. During myomectomy, despite the application of best surgical technique, excessive bleeding can be seen [5]. Some preoperative and intraoperative approaches are used to minimize the risk of bleeding [6]. Although cervical myomas are a rare type of myoma they present both technical difficulties and a serious bleeding risk during surgery [7]. PUAE is used as a high-quality preoperative myoma treatment since it can both reduce their vascularization and bleeding during surgery [8].

PUAE is an efficacious treatment that can be chosen in cases of myoma with high risk of bleeding. Patients who do suffer heavy blood loss may experience complications such as conversion to hysterectomy, infection, transfusion reactions and a long hospital stay [9].

Mc Lucas et al. reported that 12,5% of patients who underwent only myomectomy needed to have a blood transfusion, compared with no women who underwent PUAE and myomectomy [10]. Üstünöz et al. conducted a case control prospective study and found that the group who underwent PUAE had a shorter surgery time, less blood loss, no transfusion and no hysterectomy compared with the group who had only myomectomy [11]. Schnapauff et al. published a 21-patient case series that concluded that PUAE facilitates a safe and uterus-preserving myomectomy in patients with very large or multiple fibroids [12]. Considering the location and size of myoma in the patient in the present case, the amount of bleeding during the procedure was reasonable and no transfusion was required. Perhaps most importantly (because the patient wished to retain her fertility), a hysterectomy was not necessary.

Traditionally, embolization is not used in patients who wish to maintain fertility. Nowadays, there are still concerns about embolization affecting fertility as well as obstetric complications as low birth weight, miscarriage, and prematurity [13]. Karlssen et al., in a systematic review, found a lower pregnancy rate and higher miscarriage rate after embolization than after myomectomy [14]. Another systematic review, by Homer and Saridogan, found similar rates of preterm delivery, intrauterine growth restriction and malpresentation between post-embolization pregnancies and myoma-containing pregnancies, but post-embolization pregnancies were at increased risk of miscarriage, cesarean delivery, and postpartum hemorrhage [15]. Ludwig et al. in their recent review of pregnancy and outcomes after uterine fibroid embolization found embolization may be associated with an increased risk of preterm delivery and spontaneous abortion [13]. After our patient conceived spontaneously, she did not experience any problems during her pregnancy. At the 39th week, she delivered a healthy baby via a planned cesarean delivery.

PUAE is not routinely applied but seems to be an effective and safe intervention in patients with fibroids with high bleeding risk due to location and size, even if they wish to retain their fertility.
